# The Book World of Medicine and Science

**Published:** 1896-11-14

**Authors:** 


### The Book World of Medicine and Science.

Chemical Notes and Equations. By G. H. Gemmell, F.I.C., F.C.S. (London: Baillière, Tindall, and Cox. 255 pages. Price 5s. 1896.)

These notes are chiefly intended for the use of medical students preparing for their preliminary scientific examination; while apparently drawn up on the lines required by the examining boards in Edinburgh, they practically comply with the demands of the London Conjoint Board and University of London. To some extent these notes correspond to the "Students' Aid Series," by the same publishers, although they do not form a component member. The work differs from them, however, in being not only a "cram" book for examinations, but also a means of obviating the necessity for extensive notetaking during lectures, the student's attention being therefore reserved for watching the experimental operations rather than being occupied in making as many notes as possible in the time. The book is divided into three sections: I. General Principle; II. The Elements; III. Organic; and, in each of these, those portions which are most necessary from the point of view of practical medicine are most extensively dealt with. It is certainly a little book which will commend itself to students, and supply a want that has long been felt by teachers as well as pupils.

Everybody's Guide to Chess and Draughts. By H. Peachey. 226 pages, with diagrams. Cloth, 6d.; leather, 1s. (London: Saxon and Co.)

This little work certainly supplies a want. Of large works on chess, and separate articles in books of "games and pastimes," there are enough and to spare; but their acquisition requires a certain pecuniary outlay. This new addition to Saxon's "Guide Series" is within the reach of all. Chess is certainly gaining in popularity, and with the growth which penetrates to humble spheres in life, there naturally arises a demand for an authoritative handbook to this pastime, which shall contain the many complicated rules and regulations, as well as suggestions for learning and practising the game in a scientific manner. There is a chapter on openings, another on endings, and another on problems. The great masters of the game have also a short chapter devoted to an account of their lives and methods, and another useful chapter deals with the question of clubland. Draughts occupy but a very small proportion of the author's attention; indeed, little can be written on this game, since the rules are simple, and the moves and combination too infinite for anything else than mathematical expression. Throughout the pages are written in an interesting manner, and afford pleasure as well as instructive reading for all players.

Everybody's Cycling Law. By Sidney Wright and C. W. Brown. 100 pages. (London: Saxon and Co.)

A cheap legal handbook for cyclists will be appreciated by the ever-increasing army of wheel-men and wheel-women. Ignorance of the law on this subject is, perhaps, one of the chief dangers of cycling. Now that a sixpenny guide to cycling law is to be procured at every bookstall there is no longer an excuse for dangerous ignorance. The book is divided into the following sections: I. The Purchase of the Cycle; II. On the Road; III. The Cyclist at the Inn; IV. The Carriage of Cycles. Although there is no alphabetical index, there is a summary of contents at the end of the book which should enable the reader to refer quickly for legal advice to the page on which each question is briefly but clearly dealt with. No questions regarding damage, obstruction, or other difference arising between passengers, fellow-riders, and others are likely to occur which are not fully explained and dealt with in "Everybody's Cycling Law." This member of the "Guide Series" can, like all its fellows, be conveniently and easily carried in the pocket.

Gout and Goutiness, and their Treatment. By William Ewart, M.D. (London: Baillière, Tindal, and Cox. 1896. 580 pages. 12s. 6d.)

A title so comprehensive as that adopted by Dr. Ewart carries with it a prescriptive right to traverse at will the whole gamut of medicine. It reflects, therefore, most creditably on his powers of selective discrimination that he has not availed himself of this licence beyond the limits of 580 pages. Avoiding prolixity without sacrificing the literary character of the work, Dr. Ewart has elaborated a work which will rank among the classics of medicine, and as a text-book on gout it is without a rival. As a treatise on gout one might have been better pleased with a freer expression of personal views, and the reading might have been all the lighter for the inclusion of a few clinical and descriptive pictures of the various phases of gout; but as a text-book their exclusion is so much gain.

The book is divided into ten sections, of which the follow- are the most important: (1) Theories; (2) Chemistry; (3) Morbid Anatomy; (4) Clinical Study; (5) Treatment; (6) Medical Springs; (7) Prophylaxis. Dr. Ewart has evidently recognised the difficulty of adhering strictly to the natural limitation of the various sections, and of making each complete in itself, without reiteration or redundancy. The chapter on pathology illustrates this difficulty. Only twenty-eight pages are exclusively devoted to this subject because most of the important points which belong properly to it have been dealt with under headings—"Theories," "Chemistry," and "Morbid Anatomy"—from which, indeed, they could hardly have been excluded. As we have remarked before, Dr. Ewart is not very explicit as to his personal views, and nowhere is this more apparent than when treating of the pathological problems. With regard to the direct origin of uric acid from defunct and degenerate leucocytes, as believed by Horbaczewski, he has little to say in contradiction; but this is about the only indication we can gather as to the trend of his views on this most important subject. In speaking of structural changes in "goutiness," Dr. Ewart observes that they are minute in proportion to their wide extent, and are such as not to be recognised in individual cells; they are, nevertheless, "obvious, most marked in the modification of the external epithelium, and particularly that of the nails, which show decided thinning. The same structural delicacy may be inferred to exist in the nervous system from a consideration of its functional relation to the skin," and, he might have added, from its common embryological derivation from the epiblast. This is a considerable concession to the nervous theory. With regard to treatment, Dr. Ewart's remarks are of the first importance, and, indeed, they occupy nearly half the entire pages of the work. Speaking generally as regards the results of treatment, he says, although not entirely successful, we must remember with Heberden "it should not be considered a reproach to medical men till they are permitted to attempt a cure of the disease, or until they can find gouty patients who will follow their advice." As to the curability of gout, Dr. Ewart's own answer is, "Gout is curable, but some of its worst results are not; if our ætiology is right, if gout is self-inflicted, if it is the outcome of absolute or relative inactivity, coupled with over-indulgence—in short, a manufactured product of civilisation—it is certainly preventable; it may even be cured after its onset." In the treatment Dr. Ewart does not always regard theoretical consideration of the pathology as the best guide; for instance, carbonate of soda is invaluable in many gouty conditions, and yet, according to Sir W. Roberts, its theoretical interaction with the urates is calculated to intensify rather than modify the symptoms. All sides of the question are, however, most fairly and impartially dealt with, and this new work on gout and goutiness will be read and digested by all members of the profession with a deep sense of appreciation.

